# Supplementary material for: The effectiveness of the Peyton’s 4-step teaching approach on skill acquisition of procedures in health professions education: A systematic review and meta-analysis with integrated meta-regression
Source: PeerJ. 2020 Oct 9;8:e10129. doi: 10.7717/peerj.10129 (PMC7549471; doi:10.7717/peerj.10129)
Supplement: Supplemental Information 1 [file peerj-08-10129-s001.doc]

| **Section/topic** | **#** | **Checklist item** | **Reported on page #** |
| --- | --- | --- | --- |
| **TITLE** | | |  |
| Title | 1 | Identify the report as a systematic review, meta-analysis, or both. | p. 1  Title: the effectiveness of the Peyton’s 4-step teaching approach on skill acquisition of procedures in health professions education: a systematic review and meta-analysis with integrated meta-regression. |
| **ABSTRACT** | | |  |
| Structured summary | 2 | Provide a structured summary including, as applicable: background; objectives; data sources; study eligibility criteria, participants, and interventions; study appraisal and synthesis methods; results; limitations; conclusions and implications of key findings; systematic review registration number. | p. 2  A structured abstract including the sections “Background”, “Methods”, “Results” and “Conclusion” is provided. |
| **INTRODUCTION** | | |  |
| Rationale | 3 | Describe the rationale for the review in the context of what is already known. | p. 3,4  In the introductory section we have given an overview of the context of Peyton's teaching approach. |
| Objectives | 4 | Provide an explicit statement of questions being addressed with reference to participants, interventions, comparisons, outcomes, and study design (PICOS). | p. 4,5  The aim of this review was to systematically evaluate the effectiveness of Peyton’s 4 step teaching approach on the acquisition of procedural skills in health professions education and ii) to evaluate whether studies with fewer students per teacher (i.e. the student-teacher ratio) showed a larger between group difference than studies with more students per teacher. |
| **METHODS** | | |  |
| Protocol and registration | 5 | Indicate if a review protocol exists, if and where it can be accessed (e.g., Web address), and, if available, provide registration information including registration number. | p. 5  A protocol of this systematic review was registered in the OSF registries: <https://doi.org/10.17605/OSF.IO/5UE7C>. |
| Eligibility criteria | 6 | Specify study characteristics (e.g., PICOS, length of follow-up) and report characteristics (e.g., years considered, language, publication status) used as criteria for eligibility, giving rationale. | p. 5,6  Study characteristics regarding the categories “types of studies”, “participants”, “interventions”, “comparator” and “outcomes” were specified. |
| Information sources | 7 | Describe all information sources (e.g., databases with dates of coverage, contact with study authors to identify additional studies) in the search and date last searched. | p. 5  We searched the following electronic databases for eligible studies: Medline, PsycInfo, Embase and Education Resources Information Center (ERIC). In addition, references of included studies were checked for potential eligible studies. |
| Search | 8 | Present full electronic search strategy for at least one database, including any limits used, such that it could be repeated. | Appendix 1  The full search strategy is presented in Appendix 1. |
| Study selection | 9 | State the process for selecting studies (i.e., screening, eligibility, included in systematic review, and, if applicable, included in the meta-analysis). | p. 6  Records were screened by two independent reviewers. |
| Data collection process | 10 | Describe method of data extraction from reports (e.g., piloted forms, independently, in duplicate) and any processes for obtaining and confirming data from investigators. | p. 6  One reviewer extracted relevant data into an electronic database and a second reviewer controlled the data. |
| Data items | 11 | List and define all variables for which data were sought (e.g., PICOS, funding sources) and any assumptions and simplifications made. | p. 6  The primary outcome for this review was the evaluation of procedural skills. These could be evaluated using a performance metric such as a procedure specific checklist or a global rating scale. The secondary outcome was the time needed to perform the procedure. |
| Risk of bias in individual studies | 12 | Describe methods used for assessing risk of bias of individual studies (including specification of whether this was done at the study or outcome level), and how this information is to be used in any data synthesis. | p. 6  The risk of bias was evaluated using the Cochrane risk of bias tool. A human reviewer evaluated all included studies. Evaluations were compared against a machine learning classification of the risk of bias with the application “RobotReviewer”. |
| Summary measures | 13 | State the principal summary measures (e.g., risk ratio, difference in means). | p. 7  Effect sizes were interpreted following Cohen. This means that an effect size of 0.2 was considered as small, 0.5 as medium and 0.8 as large |
| Synthesis of results | 14 | Describe the methods of handling data and combining results of studies, if done, including measures of consistency (e.g., I^2^) for each meta-analysis. | p. 7  A random effects model was used for the analysis and effectiveness was reported using standardized effect sizes (Hedges’ g) and corresponding 95% confidence intervals. The Hartung, Knapp, Sidik, Jonkmann adjustment was applied to achieve robust estimations of the treatment effect.  Statistical heterogeneity was assessed with I^2^ statistics using the guidelines presented in the Cochrane handbook for systematic reviews of interventions |

Page 1 of 2

| **Section/topic** | **#** | **Checklist item** | **Reported on page #** |
| --- | --- | --- | --- |
| Risk of bias across studies | 15 | Specify any assessment of risk of bias that may affect the cumulative evidence (e.g., publication bias, selective reporting within studies). | n.a. |
| Additional analyses | 16 | Describe methods of additional analyses (e.g., sensitivity or subgroup analyses, meta-regression), if done, indicating which were pre-specified. | p. 12  Findings from a crossover study were integrated into the meta-analysis. Because data from paired analyses were not available, we adjusted the study data based on a method described by Elbourne et al. (2002). |
| **RESULTS** | | |  |
| Study selection | 17 | Give numbers of studies screened, assessed for eligibility, and included in the review, with reasons for exclusions at each stage, ideally with a flow diagram. | p. 7  The electronic search on the databases Medline, PsycInfo, Embase and ERIC identified 482 potential eligible records. The numbers screened, assessed for eligibility and included into the review are presented within the manuscript. |
| Study characteristics | 18 | For each study, present characteristics for which data were extracted (e.g., study size, PICOS, follow-up period) and provide the citations. | p. 8.  An overview of included studies and study characteristics is presented Table 1 |
| Risk of bias within studies | 19 | Present data on risk of bias of each study and, if available, any outcome level assessment (see item 12). | p. 11,12  The risk of bias regarding all assessed risk of bias categories is presented on page 11 and 12. In addition a summary risk of bias plot is presented (Figure 6). |
| Results of individual studies | 20 | For all outcomes considered (benefits or harms), present, for each study: (a) simple summary data for each intervention group (b) effect estimates and confidence intervals, ideally with a forest plot. | p. 9-12  We have provided for each outcome (“performance” and “time needed for procedure”) simple summary data for each intervention group and effect estimates with corresponding 95%CIs. |
| Synthesis of results | 21 | Present results of each meta-analysis done, including confidence intervals and measures of consistency. | p. 9-12  For each analysis performed we have provided the effect estimate with the corresponding 95%CI. |
| Risk of bias across studies | 22 | Present results of any assessment of risk of bias across studies (see Item 15). | n.a. |
| Additional analysis | 23 | Give results of additional analyses, if done (e.g., sensitivity or subgroup analyses, meta-regression [see Item 16]). | p. 12  The findings of the sensitivity analysis are presented on page 11. For example: For the meta-analysis performance at post-acquisition, the standard error of the study decreased from 0.06 to 0.04. |
| **DISCUSSION** | | |  |
| Summary of evidence | 24 | Summarize the main findings including the strength of evidence for each main outcome; consider their relevance to key groups (e.g., healthcare providers, users, and policy makers). | p. 12 ff  We have summarised the main findings for each outcome. For example: The primary finding was that Peyton’s teaching approach was more effective than a standard teaching approach on the acquisition of procedural skills at post-acquisition testing. A small to moderate effect size was associated with this finding. |
| Limitations | 25 | Discuss limitations at study and outcome level (e.g., risk of bias), and at review-level (e.g., incomplete retrieval of identified research, reporting bias). | p. 14, 15  Several limitations are discussed within the limitation section of the manuscript. This included the analysis of potential effect modifiers, inclusion and analysis of cross-over studies within meta-analyses and the exclusion of interventions from this review. |
| Conclusions | 26 | Provide a general interpretation of the results in the context of other evidence, and implications for future research. | p. 15, 16  A general conclusion was provided.  Peyton’s teaching approach is an effective teaching approach for skill acquisition of procedural skills when faculty members are used as teachers. When peer students or student tutors are used as teachers the effectiveness of Peyton’s teaching approach is less clear. Peyton's teaching approach is more effective when small groups with few students per teacher are used.  In addition, implications for practice and research are reported. |
| **FUNDING** | | |  |
| Funding | 27 | Describe sources of funding for the systematic review and other support (e.g., supply of data); role of funders for the systematic review. | We have provided a funding statement.  The authors received no funding for this work. |

*From:*  Moher D, Liberati A, Tetzlaff J, Altman DG, The PRISMA Group (2009). Preferred Reporting Items for Systematic Reviews and Meta-Analyses: The PRISMA Statement. PLoS Med 6(7): e1000097. doi:10.1371/journal.pmed1000097

For more information, visit: **www.prisma-statement.org**.

Page 2 of 2
