# Supplementary material for: The effectiveness of the Peyton’s 4-step teaching approach on skill acquisition of procedures in health professions education: A systematic review and meta-analysis with integrated meta-regression
Source: PeerJ. 2020 Oct 9;8:e10129. doi: 10.7717/peerj.10129 (PMC7549471; doi:10.7717/peerj.10129)
Supplement: Supplemental Information 2 [file peerj-08-10129-s002.docx]

## Appendix 1. Search strategy

The following strategy was used to identify studies in Medline via OVID.

| **#** | **Searches** |
| --- | --- |
| 1 | schools, medical.mp. or Schools, Medical/ |
| 2 | schools, nursing.mp. or Schools, Nursing/ |
| 3 | schools, health occupations.mp. or Schools, Health Occupations/ |
| 4 | education, professional.mp. or Education, Professional/ |
| 5 | education medical.mp. or Education, Medical/ |
| 6 | education pharmacy.mp. or Education, Pharmacy/ |
| 7 | education dental.mp. or Education, Dental/ |
| 8 | Physical Therapy Modalities/ or education physiotherapy.mp. |
| 9 | 1 or 2 or 3 or 4 or 5 or 6 or 7 or 8 |
| 10 | Peyton.mp. |
| 11 | 4 step.mp. |
| 12 | four step.mp. |
| 13 | deconstructive teaching.mp. |
| 14 | see one do one teach one.mp. |
| 15 | see one do one.mp. |
| 16 | Peyton four-step.mp. |
| 17 | Peytons four-step.mp. |
| 18 | 10 or 11 or 12 or 13 or 14 or 15 or 16 or 17 |
| 19 | resident*.mp. |
| 20 | student*.mp. |
| 21 | traine*.mp. |
| 22 | 19 or 20 or 21 |
| 23 | 9 or 22 |
| 24 | 18 and 23 |

The following strategy was used to identify studies in PsycInfo via OVID.

| **#** | **Searches** |
| --- | --- |
| 1 | schools, Medical.mp. or Schools, Medical/ |
| 2 | schools, nursing.mp. or Schools, Nursing/ |
| 3 | schools, health occupations.mp. or Schools, Health Occupations/ |
| 4 | education, professional.mp. or Education, Professional/ |
| 5 | education medical.mp. or Education, Medical/ |
| 6 | education nursing.mp. or Education, Nursing/ |
| 7 | education pharmacy.mp. or Education, Pharmacy/ |
| 8 | education dental.mp. or Education, Dental/ |
| 9 | Physical Therapy Modalities/ or education physiotherapy.mp. |
| 10 | Peyton.mp. |
| 11 | 4 step.mp. |
| 12 | four step.mp. |
| 13 | deconstructive teaching.mp. |
| 14 | see one do one teach one.mp. |
| 15 | see one do one.mp. |
| 16 | Peyton four-step.mp. |
| 17 | Peytons four-step.mp. |
| 18 | Halsted.mp |
| 19 | resident*.mp |
| 20 | student*.mp |
| 21 | traine*.mp |
| 22 | 19 or 20 or 21 |
| 23 | control:.tw. |
| 24 | random:.tw. |
| 25 | exp treatment/ |
| 26 | 1 or 2 or 3 or 4 or 5 or 6 or 7 or 8 or 9 or 19 or 20 or 21 |
| 27 | 10 or 11 or 12 or 13 or 14 or 15 or 16 or 17 or 18 |
| 28 | 23 or 24 or 25 |
| 29 | 26 and 27 and 28 |

The following strategy was used to identify studies in Embase via embase.com.

| **#** | **Searches** |
| --- | --- |
| 1 | 'medical education'/de OR 'medical education' |
| 2 | 'school'/de OR 'school' |
| 3 | 'nursing education'/de OR 'nursing education' |
| 4 | ('health'/de OR health) AND professionals AND ('education'/de OR education) |
| 5 | 'pharmacy education'/de OR 'pharmacy education' |
| 6 | 'dental education'/de OR 'dental education' |
| 7 | ('physiotherapy'/de OR physiotherapy) AND ('education'/de OR education) |
| 8 | 'student'/de OR 'student' |
| 9 | 'resident'/de OR 'resident' |
| 10 | traine* |
| 11 | #1 OR #2 OR #3 OR #4 OR #5 OR #6 OR #7 OR #8 OR #9 OR #10 |
| 12 | peyton |
| 13 | '4 step' |
| 14 | 'four step' |
| 15 | deconstructive AND ('teaching'/exp OR teaching) |
| 16 | 'see one do one teach one' |
| 17 | 'see one do one' |
| 18 | peyton AND four AND step |
| 19 | peytons AND four AND step |
| 20 | halsted |
| 21 | #12 OR #13 OR #14 OR #15 OR #16 OR #17 OR #18 OR #19 OR #20 |
| 22 | 'crossover procedure':de OR 'double-blind procedure':de OR 'randomized controlled trial':de OR 'single-blind procedure':de OR random*:de,ab,ti OR factorial*:de,ab,ti OR crossover*:de,ab,ti OR ((cross NEXT/1 over*):de,ab,ti) OR placebo*:de,ab,ti OR ((doubl* NEAR/1 blind*):de,ab,ti) OR ((singl* NEAR/1 blind*):de,ab,ti) OR assign*:de,ab,ti OR allocat*:de,ab,ti OR volunteer*:de,ab,ti |
| 23 | #11 AND #21 AND #22 |

The following strategy was used to identify studies in Education Resources Information Center (ERIC) via OVID.

| **#** | **Searches** |
| --- | --- |
| 1 | schools, medical.mp. or Medical Schools/ |
| 2 | schools, nursing.mp. |
| 3 | exp Allied Health Occupations Education/ or schools, health occupations.mp. |
| 4 | education, professional.mp. |
| 5 | education medical.mp. or Medical Education/ |
| 6 | exp Pharmaceutical Education/ or education pharmacy.mp. |
| 7 | exp Dental Schools/ or education dental.mp. |
| 8 | education physiotherapy.mp. |
| 9 | 1 or 2 or 3 or 4 or 5 or 6 or 7 or 8 |
| 10 | Peyton.mp. |
| 11 | 4 step.mp. |
| 12 | four step.mp. |
| 13 | deconstructive teaching.mp. |
| 14 | see one do one teach one.mp. |
| 15 | see one do one.mp. |
| 16 | Peyton four-step.mp. |
| 17 | Peytons four-step.mp. |
| 18 | 10 or 11 or 12 or 13 or 14 or 15 or 16 or 17 |
| 19 | resident*.mp. |
| 20 | student*.mp. |
| 21 | traine*.mp. |
| 22 | 19 or 20 or 21 |
| 23 | 9 or 22 |
| 24 | 18 and 23 |
