# Supplementary material for: The effectiveness of the Peyton’s 4-step teaching approach on skill acquisition of procedures in health professions education: A systematic review and meta-analysis with integrated meta-regression
Source: PeerJ. 2020 Oct 9;8:e10129. doi: 10.7717/peerj.10129 (PMC7549471; doi:10.7717/peerj.10129)
Supplement: Supplemental Information 3 [file peerj-08-10129-s003.docx]

**The rationale for conducting the systematic review / meta-analysis**

Acquisition of procedural skills is a key element in health professions education. Incorrectly performed procedures can cause serious adverse events in patients. Traditionally procedures are trained using a see one – do one teaching approach. More recently Walker and Peyton have reported a stepwise teaching approach (Walker & Peyton 1998). Several randomised controlled trials have been published investigating the effectiveness of this novel teaching approach. Educators in health professions education are required to use evidence-based teaching methods and are therefore in need of systematic reviews summarising the existing evidence.

**The contribution that it makes to knowledge in light of previously published related reports, including other meta-analyses and systematic reviews**

The above mentioned randomised controlled trials showed inconclusive findings. Some trials have reported findings in favour of Peyton’s approach (e.g. Balafoutas et al. 2019; Rossettini et al. 2017). Rossettini et al. (2017) showed that acquisition of a cervical mobilisation technique was considerable higher in the Peyton group compared to a standard teaching group. In contrast, Orde et al. (2010) have reported that Peyton’s teaching approach showed only minor differences on skill acquisition regarding insertion of a laryngeal mask airway at post-acquisition and retention testing compared to a traditional teaching approach. These inconsistencies should be explored with a systematic review. Furthermore, to the best of your knowledge there is no systematic review published on this topic.

**References**

Balafoutas D, Joukhadar R, Kiesel M, Häusler S, Loeb S, Woeckel A, and Herr D. 2019. The Role of Deconstructive Teaching in the Training of Laparoscopy. *JSLS : Journal of the Society of Laparoendoscopic Surgeons* 23.

Orde S, Celenza A, and Pinder M. 2010. A randomised trial comparing a 4-stage to 2-stage teaching technique for laryngeal mask insertion. *Resuscitation* 81:1687-1691.

Rossettini G, Rondoni A, Palese A, Cecchetto S, Vicentini M, Bettale F, Furri L, and Testa M. 2017. Effective teaching of manual skills to physiotherapy students: a randomised clinical trial. *Medical education* 51:826-838.

Walker M, and Peyton J. 1998. Teaching in theatre. *Teaching and learning in medical practice Rickmansworth, UK: Manticore Europe Limited*:171-180.
